# How Risky Pregnant Women Achieve Positive Antenatal Experience: A Cross‐Sectional Study of Examination of the Sense of Coherence, Prenatal Distress, and Pregnancy Acceptance

**DOI:** 10.1111/jep.70255

**Published:** 2025-08-21

**Authors:** Ayse Deliktas Demirci, Mine Oruc, Merve Kochan Aksoy, Kamile Kabukcuoglu

**Affiliations:** ^1^ Department of Obstetrics & Gynaecological Nursing, Faculty of Nursing Akdeniz University Antalya Turkey; ^2^ Department of Midwifery, Faculty of Health Sciences Antalya Science University Antalya Turkey

**Keywords:** health promotion, high‐risk pregnancy, prenatal distress, sense of coherence

## Abstract

**Rationale:**

Antenatal care incorporating a health‐promoting approach should focus on positive pregnancy experiences. This approach can bring pregnant women closer to the health dimension.

**Aims and Objectives:**

To examine the effects and explanatory power of sense of coherence and prenatal distress on pregnancy acceptance among high‐risk pregnancies.

**Methods:**

A descriptive cross‐sectional survey was conducted between April and October 2023. The sample included 219 high‐risk pregnant women who had high‐risk pregnancies and were admitted to prenatal clinics at a state hospital. *t*‐tests, ANOVA, Pearson's correlation coefficients, and hierarchical linear multiple regression were performed using IBM SPSS software version 23.0.

**Results:**

This study indicated that high‐risk pregnant women exhibited moderate levels of sense of coherence (54.43 ± 7.49), high acceptance of pregnancy (24.53 ± 5.83), and low levels of prenatal distress (11.63 ± 4.96). The research findings showed that acceptance of pregnancy was influenced by the wanted pregnancy status (*β* = 0.231, *p* = 0.002), prenatal distress (*β* = 0.275, *p *= 0.000), and sense of coherence levels of women (*β* = −0.206, *p *= 0.001). The explanatory power of the model was 34% (*F* = 5.250, *p* = 0.000).

**Conclusions:**

The research revealed a strong link between the acceptance of pregnancy and factors such as the desired status of the pregnancy, prenatal stress, and a sense of coherence. These findings highlight key psychosocial predictors of pregnancy acceptance in high‐risk pregnancies.

## 1 Introduction

Globally, the population of high‐risk pregnant women who face elevated risks for pregnancy‐related complications is expanding, which is attributed to unhealthy lifestyles, obesity [1], advanced maternal age at conception, and concomitant comorbidities [2]. High‐risk pregnancy is a matter of great significance for public health [3]. Although pregnancy is inherently associated with certain risks, high‐risk pregnancies involve unexpected pregnancy‐related medical or obstetric conditions that pose a real or potential threat to the health and well‐being of both the expectant mother and fetus. Therefore, the healthcare requirements for high‐risk pregnancies are ranked third in the World Health Organization (WHO)'s Sustainable Development Goals (SDG3) [4].

High‐risk pregnant women often experience stress and anxiety as a result of negative emotions such as unease, apprehension, and lack of control [3, 5]. High‐risk pregnant women who have difficulty accepting their pregnancy may experience challenges in adjusting to motherhood and forming a strong bond with their fetus, which can negatively impact the bonding process [6, 7]. The acceptance of pregnancy encompasses the adaptive responses of women to the physiological and psychological transformations experienced during pregnancy. High‐risk pregnant women face significant challenges in adjusting to pregnancy and motherhood, which are compounded by increased fears related to childbirth, higher rates of intrapartum complications, and lower gestational age at birth [8]. Therefore, it is crucial to explore how high‐risk pregnant women facing risks come to accept their pregnancies.

Lederma and Weis [9] indicated that the acceptance of pregnancy encompasses factors such as intentions, desires, whether the pregnancy was planned or unplanned, its timing, and whether it was wanted or unwanted. Elements influencing acceptance include whether the pregnancy was both planned and desired, the emotional state of the pregnant individual (happy or depressed), the extent of discomforts and how they are managed, responses to physical changes, and feelings of ambivalence as the due date approaches. Unplanned and unwanted pregnancies are linked to delayed prenatal care, poor maternal mental health during and after pregnancy, weak maternal–fetal attachment, dissatisfaction in relationships, inadequate social support from partners and family, poor quality of the mother–child relationship, and children experiencing mental and physical health issues and academic challenges. The role of financial stability in the acceptance of pregnancy is also discussed. Furthermore, research findings indicate that factors such as socioeconomic status, relationships, and social‐medical conditions are linked to the acceptance of pregnancy [10]. However, health‐related issues and complaints about pregnancy do not appear to influence this acceptance [10, 11]. Although the term “acceptance of pregnancy” has been used in a limited number of studies concerning high‐risk pregnancies, it is suggested that it could serve as an indicator of quality of life for pregnant women [12, 13]. Nonetheless, acceptance of pregnancy tends to be lower in high‐risk pregnancies [12, 14]. Consequently, it is essential to examine the factors influencing pregnancy acceptance in high‐risk cases, which forms the basis of hypothesis 1 in the present study.

The international guidelines recommend close monitoring and observation of maternal and fetal parameters, which may necessitate hospitalization [15, 16, 17]. Hospitalization is a critical event that presents both the physical stressors of pregnancy and the emotional and psychological challenges of hospitalization [18]. They may face prenatal distress caused by hospitalization and the compounded challenges of being away from home and family, undergoing tests and treatments, and grappling with uncertainty [19]. Prenatal distress, which is described as an emotional reaction to pregnancy and stress specific to pregnancy, may pose a threat to both the health and well‐being of high‐risk pregnant women and their infants [20], as well as negatively impact maternal‐fetal attachment [6], making it challenging for high‐risk pregnant women to adjust to pregnancy [3]. It is recognized that mental health impacts women's acceptance of pregnancy to varying degrees [10], which forms the basis for the second hypothesis of the current study. The medical aspects of high‐risk pregnancies are well documented, but further research is needed to fully understand their emotional and psychological impact on high‐risk pregnant women. WHO [21] emphasizes the importance of positive pregnancy experiences and advocates integrating antenatal care with a health‐promotion approach to achieve this goal. Therefore, healthcare professionals should evaluate high‐risk pregnant women’ resources to manage their emotional and psychological health and encourage them to recognize the resources available to them and how they can utilize them effectively.

## 2 Background

Sense of coherence (SOC) is a concept that reflects the degree to which individuals cope with stress factors [22] and comprises the concepts of comprehensibility (the individual's ability to comprehend the situation), manageability (the individual's ability to obtain and make use of adequate resources), and meaningfulness (the individual's ability to define and make sense of the world). It attempts to explain why some individuals remain healthy in the face of life events that cause stress, while others do not [23]. A strong SOC helps individuals cope with stressors and successfully manage tension [24, 25].

Women with higher SOC have better mental health and are less anxious [26]. SOC attempts to explain why certain individuals can withstand high levels of stress, remain healthy, and enable high‐risk pregnant women to understand and manage such situations by using the resources available to cope with stress [25]. Physiological, biological, and anatomical changes during pregnancy narrow the line between health and disease and pose a potential risk to the health of the mother and fetus [27, 28]. SOC is defined as a protective factor for high‐risk pregnant women and helps them approach pregnancy, childbirth, and motherhood more calmly [26]. It is highlighted that high‐risk pregnant women can demonstrate resilience by possessing internal resources, such as personal strengths like optimism, and external resources, including strong marital relationships, perceived social support, and higher socioeconomic and educational backgrounds, which offer protective benefits for both maternal and infant outcomes [29]. It is suggested that pregnant women facing the risk of adverse birth outcomes due to high chronic stress are more likely to effectively cope and adapt to their circumstances if they have access to these resources [30]. It is also noted that individuals have resources that help them manage various stressful life events and may develop a strong SOC [22]. Therefore, it is acknowledged that the SOC level of high‐risk pregnant women can influence their ability to handle risky situations and could be a resource for acceptance of pregnancy in the high‐risk pregnant women, which may be facilitated by reducing the impact of socio‐demographic, obstetrical variables, and prenatal distress levels. This understanding forms the foundation for the third hypothesis of the present study.

The significance of SOC during pregnancy lies in its ability to bring individuals closer to the health dimension, making its impact during this period a crucial aspect to consider [31]. According to WHO [21], a positive antenatal experience involves maintaining both physical and sociocultural normality, ensuring a healthy pregnancy for both mother and baby by preventing or addressing risks, illnesses, and mortality, facilitating a smooth transition to a positive labor and birth, and achieving a fulfilling motherhood experience, which includes maternal self‐esteem, competence, and autonomy. This approach prioritizes person‐centered health and well‐being, aligning with a human rights‐based perspective, rather than solely focusing on preventing death and morbidity. It is highlighted that a positive pregnancy experience should be supported by relevant and timely information, which serves as a resource for comprehensibility, a subdimension of SOC, as well as psychosocial and emotional support, which are resources for manageability, another subdimension of SOC [32]. Therefore, it is believed that SOC might act as a resource to foster a positive antenatal experience for women by promoting acceptance of pregnancy and reducing the impact of prenatal distress. However, few studies have focused on SOC as a resource for high‐risk pregnant women [24]. To the best of our knowledge, no study has examined the effect and explanatory power of SOC level of high‐risk pregnant women who have high‐risk pregnancies on acceptance of pregnancy which is a required element for antenatal adaptation. This study aims to identify the effects and explanatory power of SOC and prenatal distress on pregnancy acceptance among high‐risk pregnant women with high‐risk pregnancies admitted to the perinatology unit. Therefore, this study explains ways that present how high‐risk pregnant women with high‐risk pregnancies can be supported to have positive antenatal experience. The hypotheses of this study were as follows:

Acceptance of pregnancy in the high‐risk pregnant women is influenced by some sociodemographic and obstetrical factors.

Prenatal distress has a significant impact on acceptance of pregnancy in the high‐risk pregnant women.

SOC has a significant impact on acceptance of pregnancy in the high‐risk pregnant women.

## 3 Methods

### 3.1 Study Design

The research was carried out using a cross‐sectional and descriptive design, adhering to the Strengthening the Reporting of Observational Studies in Epidemiology (STROBE) checklist for cross‐sectional studies throughout the study [33] (Appendix S1).

### 3.2 Setting

The study was conducted in Antalya, Türkiye from April to October 2023. Eligible participants were recruited from the perinatology unit of a large state hospital in the southern region of Türkiye. This hospital is the largest state‐run medical facility in the city and has a specialized unit for high‐risk pregnancies, which is the only such unit in the area. High‐risk pregnant women who are at risk for their own health and/or that of their babies are admitted to this unit, where they and their infants are closely monitored by medical professionals. The level of activity restrictions imposed on these persons is determined by their obstetric risk level, as assessed by doctors. However, they are not provided with mental, social, or occupational pursuits.

### 3.3 Sample

The research design employed convenience sampling of high‐risk pregnant women who met the inclusion criteria, which consisted of (a) having a high‐risk pregnancy diagnosed by an obstetrician and hospitalized at the perinatology unit, (b) having live fetus/fetuses, (c) expressing willingness to participate in the study, and (d) possessing the ability to communicate and comprehend the Turkish language. Women with high‐risk pregnancies that ended in ectopic pregnancy or elective abortion were excluded from the study. A pregnancy is classified as high risk when there is an actual or potential threat to the health or well‐being of the mother and fetus, or when an unexpected medical or obstetric condition arises during pregnancy [34].

The sample size was determined using G*Power version 3.1.9. The multiple regression model analysis comprised 16 variables, 14 of which were potentially confounding variables and 2 were independent variables. To obtain a medium effect size of 0.15 with a significance level of 0.05 (two‐sided) and a power of 95%, a minimum sample size of 204 was required. Considering the potential dropout rate, 220 individuals were recruited to complete the questionnaire, resulting in a final study sample of 219 participants, with one participant not completing the study. According to Kendall's principle, the recommended sample size is at least 5–10 times the number of variables in the analysis [35]. In this study, which involved 16 variables in the final regression analysis, the estimated sample size ranged from 80 to 160. Therefore, a sample size of 219 was sufficient for the hierarchical linear multiple regression analysis. A total of 240 high‐risk pregnant women were approached to participate; however, only 219 women completed the survey.

### 3.4 Data Collection

High‐risk pregnant women were recruited from a state hospital's prenatal clinic in Antalya, Türkiye through convenience sampling, and the participants were interviewed in person, with an average interview duration of 15 min. High‐risk pregnant women were recruited face‐to‐face by the researchers (ADD, MO, MK) in the unit. The researchers explained the survey to the eligible participants before obtaining informed consent. Participants were required to complete the questionnaires during the interviews.

### 3.5 Measures

Four instruments were used during the data collection process, as detailed below.

The personal form collects information on sociodemographic and obstetric characteristics. Sociodemographic details include educational attainment, age, employment status, financial situation, and family structure [10], while obstetric details encompass the number of pregnancies, gestational age, instances of perinatal loss, and whether the pregnancy was desired and planned [9, 36], which can influence a woman's acceptance of her pregnancy. In terms of obstetric characteristics, a planned pregnancy is defined as the intention to conceive, cessation of contraception, mutual agreement with a partner, and timing that aligns with lifestyle or life stage. An unwanted pregnancy is a term used only after conception has occurred and may be linked to dissatisfaction with the pregnancy or not wanting the child [37].

The Sense of Coherence (SOC) Scale comprises 13 items with three subdimensions: meaningfulness, comprehensibility, and manageability [22]. A high score on the SOC scale indicates strong SOC. Turkish version was validated by Scherler and Lajunen [38]. Participants were asked to rate each item on a scale from 1 to 7. The test uses these ratings to indicate the probability of effective coping mechanisms and perceived mental well‐being, with scores ranging from a minimum of 13 to a maximum of 91. This study demonstrated an overall Cronbach's α coefficient of 0.69. The present study supports the use of a one‐factor model, consistent with findings from Ferguson et al. [39]

The acceptance of pregnancy sub‐dimension of the Prenatal Self‐Assessment Scale developed by Lederman et al. [40] was used to measure the adjustment of high‐risk pregnant women to pregnancy and motherhood. A study conducted by Beydağ and Mete [41] validated the dependability and accuracy of this scale in Turkish. This study uses a 14‐item scale. The minimum and maximum scores for this subscale are 14 and 56 points, respectively. A low score suggests a high level of adaptation to pregnancy. In this study, the subscale's Cronbach's alpha coefficient was *α* = 0.73.

The Prenatal Distress Scale was initially developed by Yalı and Lobel [42], comprised 12 items, and was subsequently revised by Lobel [31]. This revised version was adapted into Turkish by Yuksel et al., [43] resulting in the Prenatal Distress Scale‐Revised Version, which consists of 17 items. It employs a triple Likert‐type scale, allowing for a minimum score of 0 and maximum score of 34. A higher total score on the scale indicates a higher perceived level of prenatal distress among high‐risk pregnant women. Cronbach's alpha coefficient was evaluated in this study as *α* = 0.70.

### 3.6 Analytic Procedure

The analysis of individual characteristics involved descriptive statistics. Categorical variables from the sociodemographic data were described using frequency and percentage, while continuous variables were described using mean and standard deviation (SD). Additionally, the internal consistency of all measurements was assessed using Cronbach's alpha (α).

Before running the regression model, we tested several assumptions, including normality, multicollinearity, linearity, and homoscedasticity. It was determined that the data had a normal distribution, as evidenced by a *p*‐value of ≥ 0.05 from the Kolmogorov‐Smirnov/Shapiro‐Wilk test, and skewness within the range of −1.5/+1.5 and kurtosis within the range of −1.5/+1.5. Moreover, the data exhibited linearity with a correlation coefficient of < 0.25, and there were no multicollinearity issues, as the values were greater than 0.85, and the VIF values were less than 5. To evaluate the relationship between SOC, prenatal distress, and acceptance of pregnancy, a Pearson's correlation test was performed.

A hierarchical linear multiple regression analysis was performed to assess the impact of multiple factors on pregnancy acceptance. The “enter” method was utilized in the analysis, and the first step involved incorporating variables that showed statistical significance in the socio‐demographic and obstetric characteristics difference test. Continuous variables such as women's age, gestational age, partner's age, number of pregnancies, pregnancy loss, and living child were considered as such, while the nominal and ordinal variables that remained were converted into dummy variables. In the second step, prenatal distress was added; in the third step, SOC was included. The threshold for statistical significance was set at *p* < 0.05. Data analysis was performed using SPSS version 23 (SPSS Inc., Chicago, IL, USA).

### 3.7 Ethical Considerations

The present study was approved by the regional ethics committee at the Akdeniz University in Antalya, Türkiye (Approval Date: 08.03.2023, Approval Number: KAEK‐215). Permission was obtained from each corresponding author of the outcome instruments to use them. All participants were informed about the purpose of the research and the content of the interviews. Written consent was obtained from all participants.

## 4 Results

### 4.1 Characteristics of the Sample

The average age of the high‐risk pregnant women was 28.30 ± 5.50 years. The majority (43.4%) and their partners (41.6%) completed high school. A substantial proportion (63%) of them were unemployed and perceived their economic condition as moderate. Among high‐risk pregnant women, most pregnancies (53.9%) were planned, and the majority (87.7%) were wanted. A substantial proportion (72.1%) of the women were multiparous, and the majority (72.5%) had not experienced pregnancy loss. The gestational age was 30.19 ± 5.38 years. The average number of pregnancies was 2.44 ± 1.32. The average number of pregnancy losses was 0.30 ± 0.56. The average number of living children was 1.15 ± 1.11 (Table 1).

**Table 1:** General information of high‐risk pregnant women (*N* = 219).

					Acceptance of pregnancy	
**Characteristics**	*n*	(%)	Mean	SD	M ± SD	t/F/r (p)
**Educational level**						
Primary school	72	32.9			24.22 (0.77)	0.062 (0.940)
High school	95	43.4			23.94 (0.57)	
Undergraduate and above	52	23.7			23.90 (0.65)	
**Working status**						
Not working	138	63.0			24.13 (6.27)	0.370 (0.711)
Working	81	37.8			23.83 (4.71)	
**Educational level of partner**						
Primary school	66	30.1			24.24 (7.50)	0.365 (0.694)
High school	91	41.6			23.63 (4.90)	
Undergraduate and above	62	28.3			24.37 (4.66)	
**Perception of income level**						
Low	60	27.4			24.36 (6.61)	0.801 (0450)
Moderate	138	63.0			23.69 (5.30)	
High	21	9.6			25.23 (5.88)	
**Family type**						
Nuclear family	190	86.8			24.00 (5.61)	−0.146 (0.884)
Extended family	29	13.2			24.17 (6.59)	
**Wanted pregnancy status**						
Yes	192	87.7			23.32 (5.28)	−5.116 (0.00)*
No	27	12.3			29.03 (6.42)	
**Planned pregnancy**						
Yes	118	53.9			22.64 (5.73)	−3.984 (0.00)*
No	101	46.1			25.64 (5.33)	
**Gravida**						
Primigravida	61	27.9			22.32 (5.41)	−2.764 (0.006)*
Multigravida	158	72.1			24.68 (5.74)	
Age			28.30 (17–51)	5.50		0.033 (0.629)
Gestational age			30.19 (8–39)	5.38		−0.117 (0.084)
Number of perinatal loss			0.30 (0–3)	0.56		0.126 (0.064)
Number of living child			1.15 (0–7)	1.11		0.288 (0.00)*
**Overall score of the acceptance of pregnancy**					24.53 ± 5.83	
**Overall score of prenatal distres**					11.63 ± 4.96	
**Overall score of sense of coherence**					54.43 ± 7.49	

Factors associated with acceptance of pregnancy, prenatal distress, and SOC among high‐risk pregnant women who were admitted to the unit were analyzed. The mean scores for acceptance of pregnancy and prenatal distress were 24.53 ± 5.83 and 11.63 ± 4.96, respectively, while the mean score for SOC was 54.43 ± 7.49. Table 1 displays the differences in these factors among participants with different characteristics. The acceptance of pregnancy level was found to be significantly different based on the variables of wanted pregnancy (*p* = 0.000), planning of pregnancy (*p* = 0.000), parity (*p* = 0.006), number of pregnancies (*p* = 0.000), and living children (*p* = 0.000). Acceptance of pregnancy was significantly higher among participants with wanted pregnancies than among those with unwanted pregnancies, among those with planned pregnancies than among those with unplanned pregnancies, and among those with first pregnancies than among those with more pregnancies.

### 4.2 Effects of SOC and Prenatal Distress on the Acceptance of Pregnancy

Factors affecting pregnancy acceptance were examined by conducting a first‐stage regression analysis that included variables that displayed significant discrepancies in the simple difference test. The results indicated that gestational age (*β* = −0.134, *p *= 0.044) and wanted pregnancy (*β* = 0.251, *p *= 0.001) had a significant impact on the acceptance of pregnancy among women. The model's explanatory power was 22% (*F* = 3.234, *p *= 0.000) (Table 2), which supports Hypothesis 1.

**Table 2:** Hierarchical regression results of acceptance of pregnancy as dependent variables (*N* = 219).

	Model 1					Model 2					Model 3				
Characteristics	B	SE	β	*t*	*p*	B	SE	β	*t*	*p*	B	SE	β	*t*	*p*
(Constant)	28.517	3.911		7.291	0.000	21.808	3.960	21.808	5.507	0.000	28.193	4.319		6.528	0.000
Age	−0.163	0.132	−0.156	−1.232	0.219	−0.168	0.125	−0.161	−1.342	0.181	−0.142	0.123	−0.137	−1.162	0.247
Age of partner	−0.013	0.117	−0.013	−0.108	0.914	0.035	0.111	0.037	0.317	0.751	0.059	0.109	0.062	0.542	0.589
Gestational age	−0.142	0.070	−0.134	−2.026	0.044	−0.109	0.067	−0.102	−1.626	0.106	−0.111	0.065	−0.104	−1.693	0.092
**Educationa level**															
High school	0.408	0.998	0.035	0.408	0.683	0.136	0.948	0.012	0.143	0.886	−0.234	0.932	−0.020	−0.251	0.802
Undergraduate and above	0.336	1.415	0.025	0.238	0.812	0.371	1.342	0.027	0.277	0.782	0.067	1.313	0.005	0.051	0.960
**Educational level of partner**															
High school	0.691	1.063	0.059	0.650	0.516	1.024	1.011	0.088	1.013	0.312	1.130	0.987	0.097	1.145	0.253
Undergraduate and above	1.344	1.340	0.105	1.003	0.317	1.355	1.271	0.106	1.066	0.288	1.442	1.240	0.113	1.163	0.246
**Perception of economic level**															
Moderate	−0.143	0.929	−0.012	−0.154	0.878	0.121	0.882	0.010	0.138	0.891	0.013	0.862	0.001	0.016	0.988
High	1.148	1.552	0.059	0.739	0.461	1.704	1.476	0.088	1.154	0.250	1.734	1.441	0.089	1.204	0.230
**Family type**															
Extended family	0.98	1.159	0.012	0.171	0.865	0.457	1.101	0.027	0.415	0.678	0.379	1.074	0.022	0.353	0.724
Wanted pregnancy	4.436	1.373	0.251	3.230	0.001*	4.126	1.304	0.233	3.164	0.002*	4.085	1.273	0.231	3.210	0.002*
Unplanned pregnancy	1.290	0.824	0.112	1.565	0.119	1.171	0.782	0.102	1.497	0.136	1.061	0.764	0.092	1.389	0.166
Woking status	0.460	0.957	0.039	0.481	0.631	0.299	0.908	0.025	0.329	0.743	0.086	0.889	0.007	0.096	0.923
Primiparaous	−0.140	1.186	−0.011	−0.118	0.906	0.131	1.126	0.010	0.117	0.907	0.099	1.099	0.008	0.090	0.928
Number of pregnancy	0.634	1.140	0.146	0.556	0.579	1.029	1.084	0.237	0.949	0.344	1.335	1.062	0.307	1.257	0.210
Number of living children	0.896	1.079	0.174	0.831	0.407	0.374	1.029	0.072	0.364	0.717	−0.120	1.015	−0.023	−0.118	0.906
Number of pregnancy losses	−0.002	1.294	0.000	−0.001	0.999	−0.454	1.231	−0.045	−0.369	0.712	−0.491	1.201	−0.049	−0.409	0.683
Prenatal distress	—	—	—	—	—	0.335	0.069	0.298	4.838	0.000*	0.309	0.068	0.275	4.553	0.000*
Sense of coherence	—	—	—	—	—	—	—	—	—	—	−0.132	0.040	−0.206	−3.311	0.001*
**Model Summary**	R^2^ = 0.216, Adjusted R^2^ = 0.149, R^2^ change= 0.216, F = 3.234, *p* = 0.000*					R^2^ = 0.298, Adjusted R^2^ = 0.235, R^2^ change = 0.083, *F* = 4.697, *p* = 0.000*					R^2^ = 0.335, Adjusted R^2^ = 0.271, R^2^ change = 0.037, *F* = 5.250, *p* = 0.000*				

* *p* < 0.05.

In the second‐stage regression model, the variables from the first‐stage were integrated with prenatal distress. The analysis demonstrated that wanted pregnancy (*β* = 0.233, *p* = 0.002) and the prenatal distress level of women (*β* = 0.298, *p* = 0.000) significantly influenced the acceptance of pregnancy. These findings support Hypothesis 2.

In the final stage of the three‐stage regression model, prenatal distress and SOC levels of pregnancies were incorporated as variables. The results showed that wanted pregnancy (*β* = 0.231, *p *= 0.002), prenatal distress level (*β* = 0.275, *p* = 0.000), and SOC level of women (*β* = −0.206, *p* = 0.001) significantly impacted the acceptance of pregnancy. The explanatory power of the model was 34% (*F* = 5.250, *p* = 0.000) (Table 2). These findings support Hypothesis 3.

## 5 Discussion

High‐risk pregnancies entail not only the health/well‐being of the mother but also that of the unborn child [44]. Thus, it is essential to determine the approach that promotes their antenatal period. This study aims to determine the effects and explanatory power of SOC and prenatal distress on the acceptance of high‐risk pregnancies. The study found high levels of adaptation to pregnancy, low levels of prenatal distress, and moderate levels of SOC among these high‐risk pregnant women. However, most studies have reported that high‐risk pregnant women with high‐risk pregnancies have low or moderate levels of adaptation to pregnancy [6, 30]. Adaptation to pregnancy and the changes that occur during this period may vary for each woman and her family, leading to diverse perceptions, reactions, and even adjustment problems [28]. Therefore, this study aims to explore how high‐risk pregnant women who have high‐risk pregnancies experience a positive antenatal period.

Model 1 shows that the acceptance of pregnancy among participants was influenced by gestational age and wanting of pregnancy, with an explanatory power of 22%. As pregnancy progresses, increasing stress and fear of childbirth may contribute to the growing difficulty in adjusting to pregnancy. Badakhsh et al. [3] reported that fear of pregnancy outcomes is dominant among high‐risk pregnant women who have high‐risk pregnancies. High‐risk pregnant women who had high‐risk pregnancies expressed significant concerns about the upcoming birth's difficulty, potential permanent health complications after birth, and their ability to have future normal pregnancies. Similarly, they expressed concerns about whether the infant would have a disability, a global concern among women [45].

Model 2 shows that wanting pregnancy and the level of prenatal distress in high‐risk pregnant women have an influence on acceptance of pregnancy, with an explanatory power of 30%. This supports the result that prenatal distress level, rather than gestational age, is a significant factor affecting the acceptance level of pregnancy. Women who experience problems during pregnancy have been found to have high levels of prenatal distress and a negative perception of pregnancy [46]. Brunton et al. [7] also found that stress and depression during pregnancy affect acceptance of pregnancy. Therefore, nurses caring for high‐risk pregnant women with high‐risk pregnancies must integrate interventions that evaluate and promote their psychological health.

Model 3 highlights the significant impact of wanted pregnancy, prenatal distress, and SOC level of participants on the acceptance of pregnancy, with an explanatory power of 34%. Inclusion of the SOC factor in the model increased its explanatory power in explaining the factors influencing pregnancy acceptance. SOC is a concept that reflects how individuals handle stress and is characterized as a global orientation towards stressful life events16. In the context of pregnant women, SOC is seen as a factor that aids in adapting well to their circumstances. A robust SOC allows pregnant women to manage stress effectively and influences their emotional responses to stress [47]. During pregnancy, women often reassess, re‐evaluate, and review personal aspects such as finding meaning, resources, and planning for future life management [48]. Although there is a lack of studies specifically investigating the impact of SOC and its dimensions on the acceptance of pregnancy among high‐risk pregnant women, this process further clarifies the role of SOC and its dimensions in how high‐risk pregnant women accept their pregnancy. The present study supports that SOC has a single‐factor model, aligning with Ferguson et al. [39] This indicates that for certain groups, like high‐risk pregnant women, SOC might be more effectively understood and assessed as a single construct rather than separate dimensions. The practical benefit of using a one‐factor model is that interventions can concentrate on enhancing an individual's overall SOC, rather than focusing on each subcomponent separately. This strategy could be more practical and significant in clinical environments, where a general SOC can inform care strategies aimed at boosting women's resilience, emotional regulation, and cognitive processing during high‐risk pregnancies. Nonetheless, further research employing confirmatory factor analysis across various populations is necessary to confirm the one‐factor structure and to assess whether specific components have different predictive values in different contexts.

The wanting of pregnancy, which is a statistically significant variable in all models attempting to explain pregnancy acceptance, is related to the concept of SOC. SOC has a sub‐dimension, which is defined as a motivational dimension, “meaningfulness,” that is described as “perception of how meaningful the individual sees the negative situations in their life struggle” [22]. Therefore, wanted pregnancy could be a motivational factor for women's acceptance level to pregnancy. Nurses need to clarify the meaning of high‐risk pregnancy experiences that enable them to provide good care for maternal adaptation [36]. Nurses could be used to make sense of their pregnancies, evaluate their motivational level, and enhance their motivation for their pregnancy. The motivational dimension, which is the meaning of having high‐risk pregnancies for them, should be handled by discussion of meanings and promoted by making sense of their experience.

SOC also has a sub‐dimension, “manageability,” which refers to the instrumental or behavioral dimension, which is the extent to which an individual feel that they have the resources necessary to address the demands of the stimuli they are subjected to [49]. High‐risk pregnancy was identified in a systematic review as negatively affecting coping, well‐being, and psychopathology, and they emphasized the importance of women being able to keep their pregnancies under control to alleviate this [50]. However, it is also known that high risk pregnant women mostly have high level of prenatal distress levels [51, 52, 53]. It is suggested that prenatal distress level of pregnant women which have an impact on the acceptance of pregnancy at the high‐risk pregnant women, as shown in the present study may predict by the hospital admission, and SOC levels [25]. Therefore, SOC levels might offer a way to adapt to these challenges by fostering a sense of manageability, although further research is necessary.

The final dimension of SOC, comprehensibility, which is a cognitive factor, is determined by the degree to which stimuli from both internal and external sources are perceived as logical, coherent, and well‐organized, rather than chaotic, disordered, and random. This dimension of rational understanding is crucial for processing information that is clear, structured, and easily comprehensible [49]. In the interventional study conducted by van den Heuvel et al. [54] the uncertainty of high‐risk pregnancy remains an intense experience for both women and their families. Therefore, nurses should present caring interventions that provide feelings of comprehensibility to high‐risk pregnant women by providing information about their real situations.

In the final model, factors such as pregnancy week, planned pregnancy status, and parity, which are initially influential in pregnancy acceptance, are insignificant. On the other hand, wanted pregnancy, stress, and SOC levels have saved their significance in the acceptance of pregnant women. This demonstrates the role of SOC in independence from the influence of socio‐demographic and obstetrical variables and in providing resources for achieving health and well‐being. The social determinants of health are important drivers of maternal mortality and morbidity. In a systematic review by Wang et al., [55] the roles of race and ethnicity, insurance, and education in pregnancy‐related mortality and severe maternal morbidity risks are accepted. However, this study highlights an important pathway for individuals who are not equally positioned in terms of health determinants to reach health status by overcoming the influence of existing demographic and obstetrical factors. SOC may enable high‐risk pregnant women to have positive antenatal experiences by ensuring low levels of prenatal distress and high levels of pregnancy acceptance. Therefore, nurses need to be aware of the positive effects of SOC on reducing the increased psychological burden of high‐risk pregnant women’ health status, who are vulnerable due to high‐risk pregnancies.

### 5.1 Limitations

This study has several strengths. First, the sample group was characterized by increased vulnerability due to high‐risk status. Second, the study also attempted to present SOC as a resource for high‐risk pregnant women to have positive antenatal experiences. However, this study had certain limitations. First, the sample group consisted primarily of multiparous and highly educated high‐risk pregnant women, but these variables were not identified as statistically significant factors influencing the acceptance of pregnancy levels. Additionally, the study was conducted at a single center.

## 6 Implications

This study sheds light on the impact of wanted pregnancy status, prenatal distress, and SOC levels on the acceptance of pregnancy among high‐risk pregnant women, which could potentially lead to better adaptation to their pregnancy. To promote wanted pregnancy status, interventions should focus on increasing maternal attachment and providing communication with the baby. Additionally, prenatal distress can be reduced by elevating women's SOC levels, which has already been demonstrated to affect the acceptance of pregnancy. Owing to the dimensional nature of SOC, interventions that enhance women's manageability, meaningfulness, and comprehensibility are necessary. To improve women's adaptation to pregnancy, nurses who care for high‐risk pregnant women can provide emotional support and clarify their feelings about having high‐risk pregnancies. It is crucial to ensure that women have access to visible resources to manage their problems and to identify additional resources. Moreover, it is vital to provide enough information to high‐risk pregnant women to provide comprehensibility (what is the real situation that they experienced) to high‐risk pregnant women. Experiences of consistency, which refer to the extent to which messages are clear in an individual's life, provide the basis for the comprehensibility component of the SOC. Therefore, nurses need to interact with high‐risk pregnant women who have clear messages and sufficient information about them. By doing so, women can have positive antenatal experiences with the aid of SOC. Additionally, policy and program development should focus on ensuring that resources are visible and accessible, and that communication strategies deliver consistent and clear information to enhance positive prenatal experiences.

## 7 Conclusions

This study demonstrates that women facing high‐risk pregnancies exhibit moderate levels of SOC, couple with high acceptance of pregnancy and low levels of prenatal distress. Women's acceptance of pregnancy is influenced by their pregnancy status, prenatal distress, and SOC levels. It is worth noting that wanted pregnancy, which has been established as a motivational factor for SOC, and prenatal distress, which is known to be alleviated by SOC, are both variables related to SOC. It is also demonstrated the role of SOC in independence from the influence of socio‐demographic and obstetrical variables and in providing resources for achieving health and well‐being. This finding is particularly encouraging, as it suggests that SOC may help reduce the influence of health determinants and promote equity in pregnancy health and well‐being. Although “SOC is recognized as a valuable resource that enhances the acceptance of pregnancy by mitigating the impact of other sociodemographic and prenatal factors” in the present study, this finding needs to be examined in the different populations which have different backgrounds such as race, religion, education, insurance status, marital status etc. There is also need to examine in which mechanisms SOC contribute to the antenatal positive experience by using not only quantitative but also qualitative designs in the studies. From a practical implication, it is essential for healthcare providers, particularly nurses, to be trained in evaluating and supporting SOC levels in pregnant women who are at high risk. Incorporating techniques that enhance SOC into standard prenatal care—such as structured counseling, tailored education, clarification of meanings and ongoing emotional support—can lead to improved adaptation outcomes.

## Conflicts of Interest

The authors declare no conflicts of interest.

## Data Availability

The data that support the findings of this study are available from the corresponding author upon reasonable request.
